# Different bone sites-specific response to diabetes rat models: Bone density, histology and microarchitecture

**DOI:** 10.1371/journal.pone.0205503

**Published:** 2018-10-22

**Authors:** Yunwei Hua, Ruiye Bi, Yue Zhang, Luchen Xu, Jiaoyang Guo, Yunfeng Li

**Affiliations:** State Key Laboratory of Oral Diseases, National Clinical Research Center for Oral Diseases, West China Hospital of Stomatology, Sichuan University, Chengdu, China; Rensselaer Polytechnic Institute, UNITED STATES

## Abstract

**Background and purpose:**

Diabetes mellitus (DM) is the most common metabolic disorder that is characterized by hyperglycemia, it can be categorized by T1DM and T2DM. T1DM is also reported to cause bone loss. However, most reports regarding this aspect of T1DM have only investigated a single site; a comparison of bone loss from different areas of the body is still lacking.

**Methods:**

Thirty-five 12-week-old Sprague Dawley® (SD) rats were separated to seven groups. Five rats were euthanized without any surgery at 0 weeks for histological examination and determination of baseline characteristics. In 15 of the rats, DM was induced via Streptozotocin (STZ)-injection, and they were separated to 3 groups (4 weeks, 8 weeks and 12 weeks after STZ-injection). The remaining 15 rats were used as the control group (4 weeks, 8 weeks and 12 weeks after saline-injection). We tested bone-mass loss at four skeletal sites, the tibia, the femur greater trochanter, the spine, and the mandibular bones using micro-computed tomography (CT) and histological tests.

**Results:**

Tibia was influenced the most obvious(BV/TV decreased by 27.3%, 52.5%, and 81.2% at 4 weeks, 8 weeks, and 12 weeks, respectively. p<0.05). In contrast, the other three sites were influenced to a lesser extent and bone loss became prominent at a later time point according to the histological and micro-CT tests(Femur: BV/TV did not decrease significantly at the first month or second month. However, and decreased by 49.4% at the third month, P<0.05. Mandible: the BV/TV only decreased by 6.5% at 1 month after STZ-injection. There was still a significant difference between the second and third months. The BV/TV decreased by 47.0% and 68.1% at 2 months and 3 months, respectively, (p<0.05) Spine: the BV/TV only decreased by 6.7%. However, significant change was observed in the spine at the second month and third month after STZ injection. The BV/TV decreased by 45.4% and 64.3%, respectively, p<0.05).

**Conclusion:**

The results indicate that T1DM can severely influence the bone structure of the 4 skeletal sites. Further, areas with dense trabecular bones were influenced less and at a later time point in comparison to the tibial region.

**Clinical relevance:**

Our research can serve as a guide to help increase the success rate of implant treatment, and help decrease the fracture risk in different bone types with greater accuracy.

## Introduction

Diabetes mellitus (DM) is the most common metabolic disorder characterized by hyperglycemia and associated with many diseases, such as retinopathy, nephropathy, cardiovascular disease, and osteoporosis [1]. In 2012, the prevalence of diabetes in adults between the ages of 20 and 79 years worldwide was estimated at about 382 million persons, and it was likely to increase to 592 million people by 2035.[2] In the United States of America (USA), the prevalence of DM has reached 10.9%. Approximately $101.4 billion (uncertainty interval [UI], $96.7–106.5 billion) was spent on diabetes in the USA, including 57.6% spent on pharmaceuticals and 23.5% (UI, 21.7–25.7%) spent on ambulatory care, which was the highest health care expense in 2013 [3]. China also has a large health burden of diabetes: in 2013, a quarter of diabetic patients worldwide were in China, where 11.6% of adults had diabetes and 50.1% had prediabetes.[4] DM can be divided into type I diabetes (insulin-dependent) and type II diabetes (non-insulin-dependent). Both of these 2 kinds of DM can cause hyperglycemia and several chronic bone metabolic diseases, including diabetic osteoporosis (DOP).[5]

About 1/3 to 1/2 of diabetic patients have decreasing bone strength and increasing fracture risks, and nearly 1/3 of them are diagnosed as having osteoporosis [6]. Past literatures have reported that both T1DM and T2DM can cause bone mineral density(BMD) decreased, and the negative effect of decreasing BMD is the higher risk of fracture.[7] However, the decrease of BMD at each site is different [8]. A clinical study showed that the risk of hip fracture in patients with T1DM was 6 times higher than that in healthy people (mean age, 65 years) while T2DM was 2.5 times higher than health people[9]. It was also found that patients with DM had an increased risk of fracture of the wrist and hip. In other areas, such as the spine, it also seemed that fracture occurred more frequently in populations with DM than in healthy people [10]. With the increased number of patients with DM, DOP has become a worldwide health burden.

Previous studies about ovariectomized (OVX) osteoporosis have confirmed that long bones and the spine are more susceptible to osteoporosis than the jaw bones[11]. This might be due to their different morphology and structure. However, differences of the bone strength and structural changes between different sites of bones in patients with DOP have still not been studied systematically. To illuminate this question, we used a streptozotocin (STZ)-induced rat model as our research object and micro-computed tomography (CT) and histology testing to investigate the bone mass and bone micro-structure in different areas.

Many studies have concentrated on osteoporosis and some of its related fields, such as implant osseointegration[12], the methods of anti-osteoporotic therapy[13], and bone fracture healing [14] in osteoporotic patients. Bones in different areas have different characteristics. This might be the reason why DOP affects different sites of bones differently. Through this research, we may be able to determine a regional difference of bone loss by Micro-CT and histology tests in rats with DOP.

The aims of this study are as follows (1) to research the effect of T1DM on bone mass and the bone micro-architecture at 4 skeletal sites and at different time points; (2) to investigate the difference of bone loss in 4 skeletal sites at different time in our proven STZ-induced rat model.

## Materials and methods

### Animals

All animals’ care and use were conducted in accordance with the same international standard. Our study conformed to the Animal Research Committee of the West China Hospital of Stomatology, Sichuan University, and the Ethical and legal approval was obtained from Research Ethics Committee of West China Hospital of Stomatology, Sichuan University, prior to the commencement of this study. Thirty-five male adult Sprague-Dawley rats aged 12 weeks and weighing 270–300 g (Dashuo, SiChuan, China) were used in this study. To observe the significant changes between diabetic rats and normal rats, we used 15 rats each group. Five rats were placed in each cage, and they were kept under climate-controlled conditions with light, humidity, and temperature(12 h light/dark cycle, 22–24°C, and 50–60% humidity). Rats had the same diet and free access to have standard food(12% calories) and water.

After 1 week of acclimation, five rats were euthanized without any surgery at 0 weeks for histological examination and determination of baseline characteristics. All others rats were randomly divided into 2 groups: the diabetic group and control group. Diabetic group was given single intraperitoneally injection with 50-mg/kg freshly prepared streptozotocin (STZ, Sigma Aldrich, St. Louis, MO, USA,)which was dissolved in 0.1M citrate buffer(PH 4.5). The control group was injected with the same volume of saline solution intraperitoneally. After 4, 8, and 12 weeks, five rats from diabetes group and control group(total of 10 rats) were euthanized.

### Blood glucose test

The blood sample was collected from the tail vein by rats tail snipping. Blood glucose was measured by Accu-Chek glucose meter (Roche Diagnostics, Canada) at day 3 and day 14 after STZ injection. Animals with non-fasting blood glucose over than 300mg/dl were considered as diabetic.[15,16]

### Micro-CT

Bone samples from 4 sites, including the mandible, spine (third lumbar vertebra), femur, and tibia, were harvested and analyzed by micro-CT and a histology test. All samples were fixed in 4% concentration of paraformaldehyde and scanned using a micro-CT system (micro-CT 50 scanner, Scanco Medical, Bassersdorf, Switzerland), and they were reconstructed with a voxel size of 20 μm. The scanning system was set to 70 kV, 114 μA, and 700 ms of integration time. After scanning, we used a 3-dimensional Gaussian filter (sigma = 1.2, support = 2) to eliminate noise in the volumes. The region of interest (ROI) only included the trabecular bones, without cortical bones, because measuring trabecular bones might be more appropriate to show the bone mass changes in T1DM patients.[17]

Four skeletal sites were chosen as the representative areas, including the mandibular bone, proximal tibia, femur greater trochanter, and spine. All 4 sites were commonly used in many bone research studies, and they had a good site-specific response in past studies. According to previous literature in OVX rats, osteoporosis affected jaw bones much less than long bones.[11] Thus, we suppose that DOP may affect areas with dense cancellous bone much less than general areas. Therefore, we chose the femur and greater trochanter as another position because these bones are comprised of dense cancellous bones.

#### ROIs

**Fig 1:**
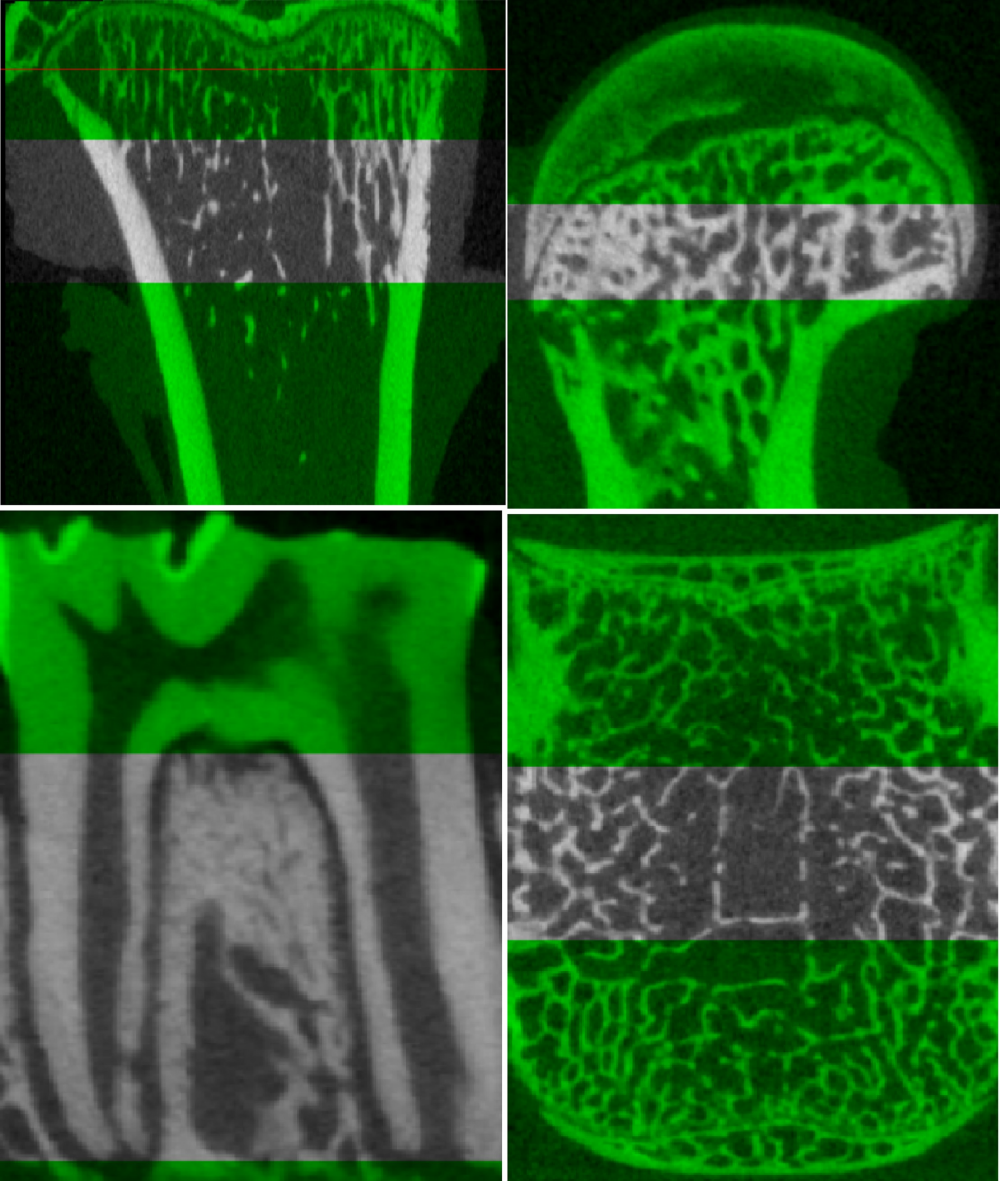
The ROI diagram of tibia, femur, mandible and spine.

All of the ROI diagrams were shown in Fig 1

Mandible: The trabecular area between the first molar root was selected as the ROI of the mandible, as a past study reported[18].

Spine: A 2-mm thickness of the trabecular bone area in the middle of the third lumber vertebral body was selected.[11]

Femur: A 1-mm thickness(50 slices) of the greater trochanter in the femur from the slice with the largest diameter in the axial plane was selected.

Tibia: A 2-mm thickness of the trabecular bone of the tibia in the axial plane (from 1 mm below the growth plate) was selected to avoid the effect of new bone growth near the growth plate[19].

#### Micro-CT analysis

For all 4 bone sites, the following indexes of the trabecular bone were chosen to evaluate the bone density and microstructure: BMD, percent bone volume ratio (BV/TV, %), trabecular number (Tb.N, /mm), trabecular separation (Tb.Sp, mm), and trabecular thickness (Tb.Th, mm), where higher values indicate reduced connectivity [20]. All these parameters can indicate the destruction of bones.

### Histologic analysis

Rats were euthanized at 0, 4, 8, and 12 weeks after the STZ injection. All samples were decalcified 3 weeks and curetted by the Leica CM3050S (Leica Microsystems AG, Wetzlar, Germany), the samples were sectioned with their maximun cross section in vertical axis and the slices were about 3μm thick, then they were stained by hematoxylin and eosin. All images were obtained the Zeiss Imager Z2 microscope (Zeiss, Vienna, Austria).

### Statistical analysis

SPSS 19.0 (IBM Corp., Armonk, NY, USA) was used in all tests to compare the measurements between the diabetic group and control group. After checked the homoscedasticity was the same, the one way-ANOVA followed by Newman-Keuls post hoc tests was used to determine significant differences. P<0.05 and P<0.01 were set to indicate a significant difference.

## Results

### Body weight

Changes in body weight are presented in Table 1. All animals in the control group had a significant increase in body weight over time, but the body weight of rats in the diabetic group did not increase. This result proved that diabetes can cause weight loss.

**Table 1:** The result of rats body weight changes in 4 weeks, 8 weeks and 12 weeks after STZ-injection.

Body weight(g)	4 weeks	8 weeks	12 weeks
DOP	245±18*	252±28*	240±23**
Control	298±16	360±20	436±19

Data were expressed as mean±standard deviation (SD).

* p<0.05 and

** p<0.01 vs. Control (ANOVA).

### Blood glucose level

The blood glucose of each rat was measured on day 3 and day 14 after STZ injection to confirm that the model was set up successfully (over 300 mg/dl). All rats in the diabetic group were in a hyperglycemic state at day 3 and they still remained diabetic at day 14. (Table 2)

**Table 2:** The result of rats blood glucose changes in 3 days and 14 days after STZ-injection.

Blood glucose (mg/dl)	3 days	14 days
DOP	336±18**	351±28**
Control	105±6	98±12

Data were expressed as mean±standard deviation (SD).

* p<0.05 and

** p<0.01 vs. Control (ANOVA).

### Micro-CT findings

**Fig 2:**
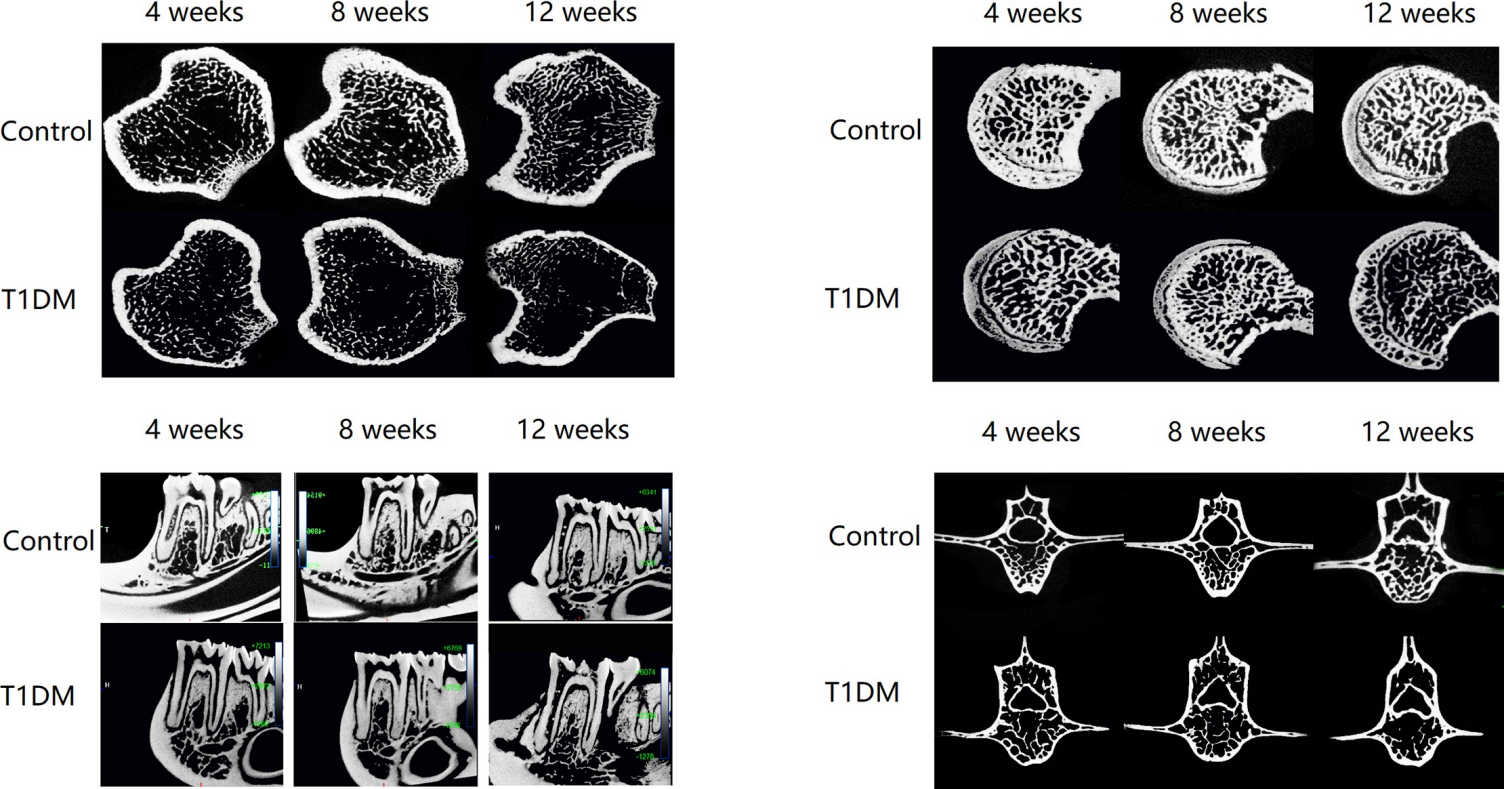
Micro-CT traverse images of ROI from tibia, femur, mandible and spine. Compared with control group and diabetes group at 4 weeks, 8 weeks and 12 weeks after STZ-injection.

Micro-CT showed that the bone micro-architechture in all 4 sites were influenced, but some of them showed some interesting phenomena, the bone micro-architechture of tibia showed significantly changes from 4 weeks to 12 weeks after STZ injection, mandible and spine changed from 8 weeks to 12 weeks, while the femur greater trochanter only changed in 12 weeks after STZ injection. The bone micro-architechture changes were shown in Fig 2.

#### ROIs

**Fig 3:**
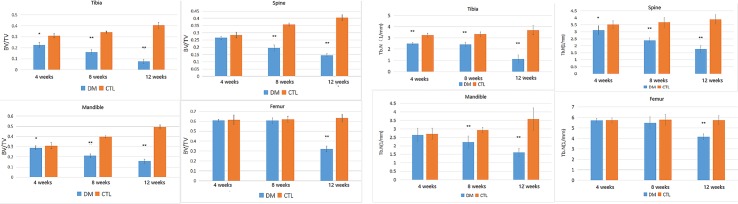
Changes in BV/TV and Tb.N of all 4 bone sites between control group and diabetes group. The parameters were expressed as mean±SD. The error bar in figure was SD. n = 5 specimens per group, * p<0.05 and ** p<0.01 vs control group.

Tibia: In our experiment, the tibia showed the most severe bone loss compared with the other areas (Fig 3). Additionally, the micro-architecture of the tibia was influenced much more severely over time (the BV/TV decreased by 27.3%, 52.5%, and 81.2%, the Tb.Th decreased by 32.1%, 48.2%, and 58.3%, the Tb.N decreased by 23.1%, 27.1%, and 69.6%, while the Tb.Sp increased by 1.53 fold, 2.6 fold, and 4.63 fold at 4 weeks, 8 weeks, and 12 weeks, p<0.05, respectively) (S1 Table).

Femur greater trochanter: A special phenomenon was found in the femur and greater trochanter in that the BMD and trabecular bone test parameters did not decrease significantly by the first month or second month (Fig 3). However, the BV/TV decreased by 49.4% at the third month compared with the control group, while the Tb.Th decreased by 22.2%, the Tb.N decreased by 27.7%, and the Tb.Sp increased by 1.3 fold at the third month.(p<0.05, respectively) (S2 Table).

**Fig 4:**
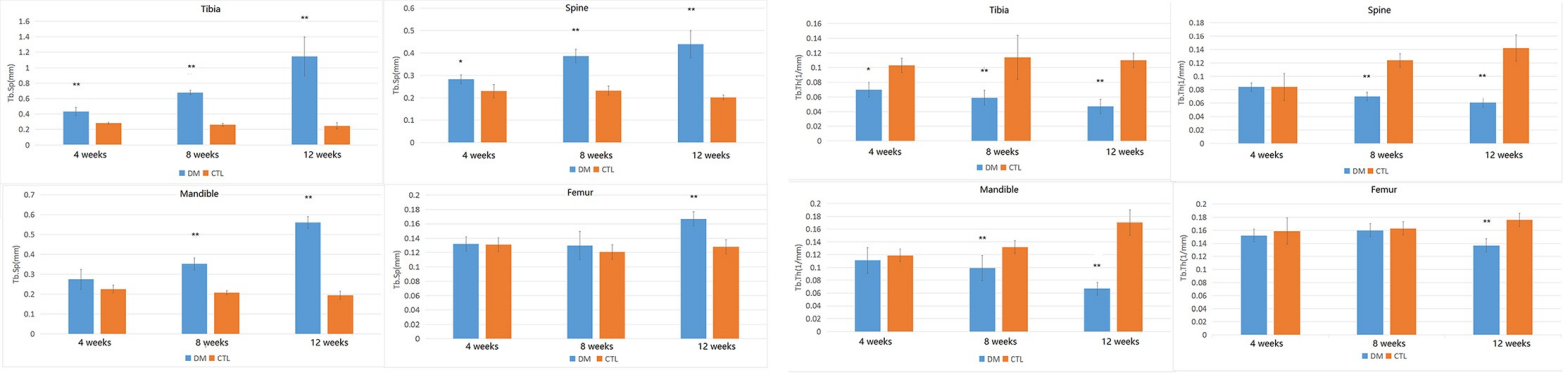
Changes in Tb.Th and Tb.Sp of all 4 bone sites between control group and diabetes group. The parameters were expressed as mean±SD. The error bar in figure was SD. n = 5 specimens per group, * p<0.05 and ** p<0.01 vs control group.

Mandibular bones: The result of mandibular bones was similar to that of the spine (Fig 4). Although the results showed a significant difference, the BV/TV only decreased by 6.5% at 1 month after STZ-injection. There was still a significant difference between the second and third months. The BV/TV decreased by 47.0% and 68.1%, the Tb.Th decreased by 25% and 61.6%, the Tb.N decreased by 35.1% and 55.8%, while the Tb.Sp increased by 1.69 fold and 2.88 fold at 2 months and 3 months(p<0.05, respectively), after STZ injection. (S3 Table).

Spine: The bone mass was barely affected in the first month, and the BV/TV only decreased by 6.7% (S4 Table). However, significant change was observed in the spine at the second month and third month after STZ injection (Fig 4). The BV/TV decreased by 45.4% and 64.3%, the Tb.Th decreased by 45% and 57%, the Tb.N decreased by 24% and 55%, while the Tb.Sp increased by 1.67 fold and 2.18 fold at the second month and the third month after STZ injection(p<0.05, respectively).

### BMD

The parameters of BMD are shown in S5 Table.

Tibia: The BMD in the diabetic group tend to indicate severe loss of bone mass, and the BMD decreased gradually in the first month(decreased by 28%, p<0.05), second month(decreased by 55%, p<0.05), and third month(decreased by 76%, p<0.05).

Femur: Results of the femur were greatly different than those of the tibia. After STZ injection, the femur BMD didn’t significantly change in the first month and second month, but showed significant decreased (decreased by 37%, p<0.05) in the third month.

**Fig 5:**
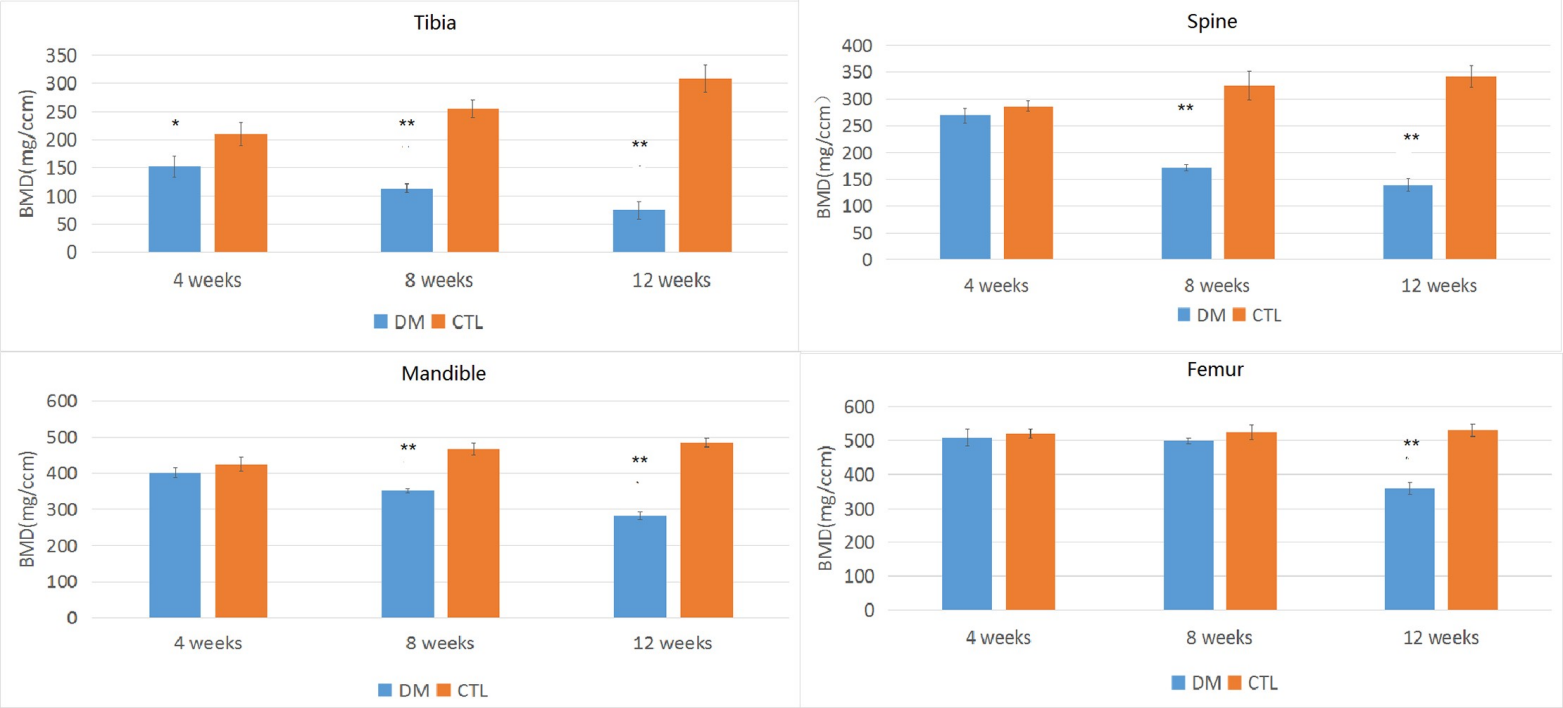
Changes in BMD of all 4 bone sites between control group and diabetes group.The parameters were expressed as mean±SD. The error bar in figure was SD. n = 5 specimens per group, * p<0.05 and ** p<0.01 vs control group.

Mandibular bones and spine: In the diabetic group, the downtrend of BMD started in the second month(spine: decreased by 47%, mandible: decreased by 25%, p<0.05, respectively), and in the third month, the downtrend was more severe than in the second month(spine: decreased by 60%, mandible: decreased by 42%, p<0.05, respectively). (Fig 5).

### Histologic findings

**Fig 6:**
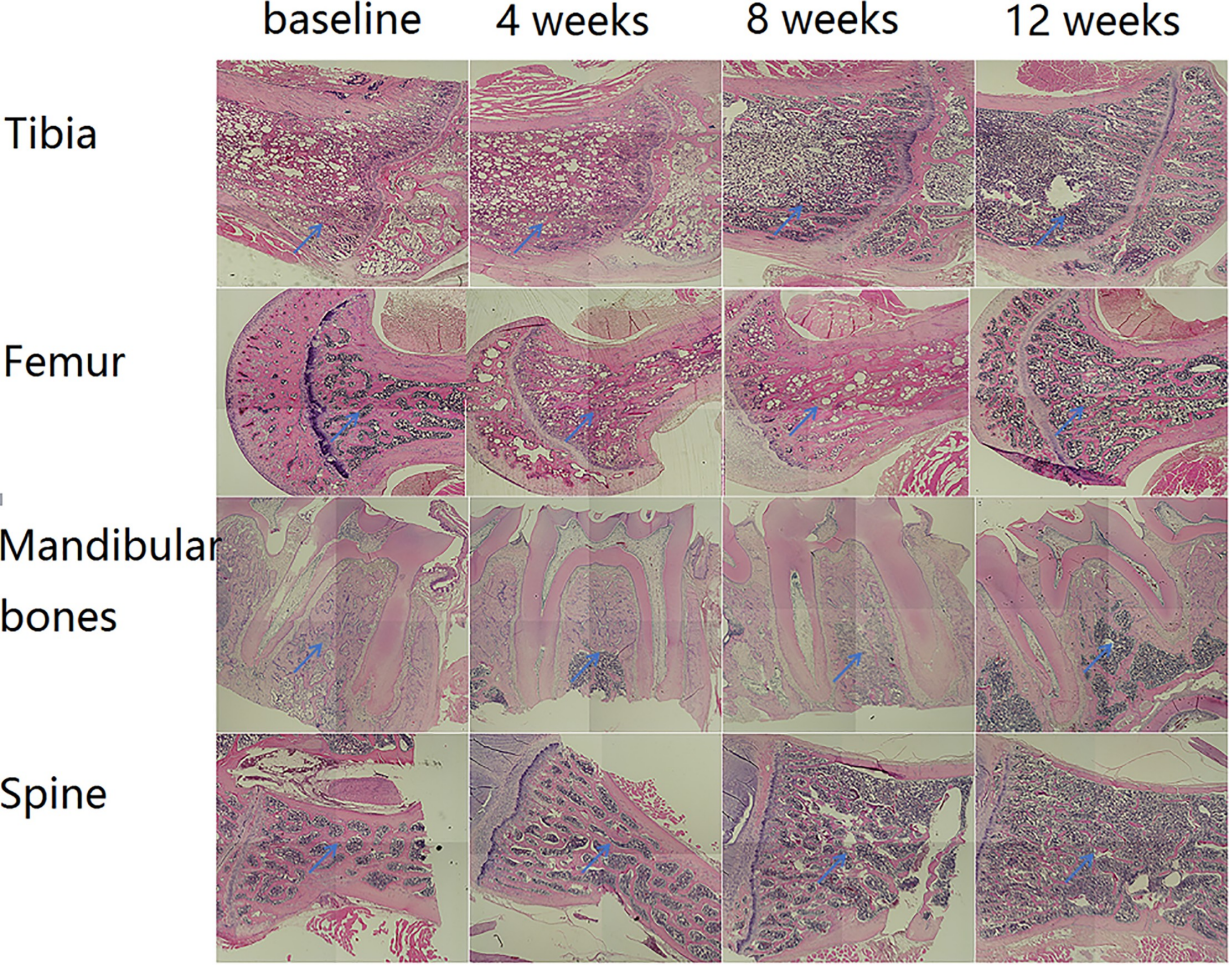
Changes in histology test of all 4 bone sites from baseline to 12 weeks after STZ- injection. The arrows show the bone micro-architechture changes.

The decalcified sections showed that the bone mass of diabetic group decreased from baseline to 12 weeks after STZ injection. The results of histology tests were similar to micro-CT findings at all 4 sites: all of the 4 bone sites in diabetic rats had significant bone loss, and they showed the phenomenon of hysteresis in femur greater trochanter, mandible and spine, but tibia didn’t show the hysteresis phenomenon. Additionally, the trabecular bones became thinner over time.(Fig 6)

## Discussion

Recent studies have reported that diabetes can affect bone quality and lead to bone loss, causing fracture and osteoporosis[21]. In addition, bone loss can also impair implant osseointegration in orthopedic and dental treatments [22]. Implant treatments can be used at many sites, such as the mandibular bones, tibia, and hip. Thus we aimed to investigate whether diabetic osteoporosis also leads to a different severity of bone loss at different bone sites. In our study, we separately clarified different phenomena of diabetic osteoporosis at different bone sites, including the proximal tibia, femur, mandibular bone, and spine, in rats with T1DM and we found that the areas with dense trabecular bones were influenced less and at a later time point in comparison to the tibial region. Moreover, our research can be used to help physicians clinically prevent fractures due to diabetic osteoporosis. It can also provide physicians with more implant treatment options for diabetic patients.

The STZ-induced T1DM rat model has been widely used in many experiments. It is a well characterized animal model to investigate the metabolism and pharmacology of diabetes[23]. T1DM always happens in young people and associated to inheritance, to imitate T1DM patients, we used growth rat to set up STZ-induced T1DM rat model. It has been reported that the injection of STZ can cause the targeted death of pancreatic B-cells, resulting in insulin deficiency and hence causing hyperglycemia in rats[24]. Many previous studies have shown that hyperglycemia can lead to bone loss, a decrease in bone density, and bone micro-architecture impairment [25,26] in rats. It may directly affect advanced glycation end products, a matter that can be stored, making bone more fragile [27]. Hyperglycemia also changes the mineral composition and collagen integrity of bone, causing the marrow cavity to become full of fat and adipogenic mesenchymal stem cells [28,29]. All of these factors lead to the inhibition of bone formation and the promotion of bone resorption, reducing the remolding of bone in diabetic rats. Besides, a past study reported that significant weight loss may cause adults to experience bone loss to some degree[30]. Moreover, Liu’s research said that body weight loss may influence weight bearing skeletal stronger than non-weight bearing skeletal.[31] So we speculated that the weight loss may be associated with the bone loss in rats with T1DM.

In our experiment, we studied changes of two main bone features in the diabetic animal model: bone mass and bone quality. BMD is an important index to assess bone mass, and it is widely used to analyze bone strength in the clinical setting. The quality of trabecular bone was reflected by MicroCT findings, including the BV/TV, Tb.N, Tb.Th, and Tb.Sp, which constituted the minimal set of variables to describe the change in trabecular bone morphometry[32].

In our study of long bones, the influence caused by T1DM was severe; BMD, BV/TV, Tb.N, and Tb.th decreased, while Tb.sp increased compared with parameters of the control group at the same time point. These results were also found in the mandibular bone; however, they only occurred in the second and third months, and the degree of these changes was less than that with the long bones. It has been confirmed that OVX osteoporosis minimally affects mandibular bones[33] compared with long bones, Mavropoulos’s research also showed that OVX influenced mandible difference in the rat with normal diet and soft diet, and they thought normal masticatory function may partially protects the rat mandibular bone from estrogen-deficiency induced osteoporosis.[34] Besides, Aghaloo’s research also showed that mandible BMSCs have a higher osteoblastic potential compared with long-bone BMSCs. Both of mechanical stimulation and the difference of bone growth potential in mandible and long bones causes the different affect in mandible and tibia.[35]

For the study of spine, the result was similar to that of the mandibular bones, which showed close parameters in the first month and a significant effect in the second and third month, when compared with the control group.

For the study of the femur, the test parameters showed that there was no obvious difference between the diabetic group and control group at 4 weeks and 8 weeks, but a significant bone loss occurred in the greater trochanter after STZ induction at 12 weeks. From this result, we can infer that DOP also influences bone mass in dense cancellous bone areas, but the effect is less and has a long hysteresis compared with the control group. It means that the negative influence of DM in dense trabecular bone area may be observed later than non-dense trabecular bone area. DM doesn’t show significant influence in dense trabecular bone area at first, but the influence in micro-architecture has started, and it may shows significant bone loss after some time. This phenomenon reminds the clinical doctor to pay more attention to dense trabecular bone areas in T1DM patients before the significant bone loss happens. Although the destruction of mandibular bones and the greater trochanter occurred later than that of the tibia and spine in the growth period of rats, the bones were ultimately influenced. Perhaps the bone formation and bone resorption remain in a state of dynamic equilibrium in dense trabecular bone areas of diabetic rats at the beginning, but this state was broken at the second month and the bone loss phenomenon was observed. And the influence from DOP occurred rapidly, the degree of this influence was also severe.

Some limitations in our study should be acknowledged. The 12 weeks observation time can reflect the changes of bone mass in the early phase. Longer observation time can be used to research the long-term changes of bone mass in T1DM rats. Besides, we used STZ-injection rats model to imitate humans. But the physiological conditions of diabetic rats and diabetic patients were different, so the different bone sites-specific response in T1DM patients still needs further research. Moreover, the lack of mechanical test may also a limitation, because of the irregular form of femur greater trochanter and mandible, the mechanical test may has significant error. Another limitations is our study doesn’t include cortical bone analysis, in reality, the bone loss of cortical bone also a reason of high fracture risk and high implant treatment failure risk of T1DM, though the bone loss of trabecular bone always shows more significant. It also needs comprehensive research in the future.

## Conclusion

We conclude that DOP affects the quality of all 4 skeletal sites, especially in long bones, and DOP also has milder and postponed effect on the mandibular bones, spine, and greater trochanter. In locations with dense cancellous bone, the effect is less than that in the areas without dense cancellous bone, and it is always accompanied with hysteresis. This information can help physicians prevent patients with DOP from developing fractures and provide more sensitively treatment options, including implants. Patients with DOP may easily develop fractures of the long bones and other sites that lack cancellous bone, and the risk of implant failure may increase at these sites. Although these patients have a diabetic disease, the success rate of implants might be comparatively higher in areas of dense trabecular bone.

## Supporting information

S1 Table Quantitative result of MicroCT test of diabetes group and control group trabecular bones mass in tibia, including BV/TV, Tb.Sp, Tb.Th and Tb.N. (DOC)

S2 Table Quantitative result of MicroCT test of diabetes group and control group trabecular bones mass in femur, including BV/TV, Tb.Sp, Tb.Th and Tb.N. (DOC)

S3 Table Quantitative result of MicroCT test of diabetes group and control group trabecular bones mass in mandible, including BV/TV, Tb.Sp, Tb.Th and Tb.N. (DOC)

S4 Table Quantitative result of MicroCT test of diabetes group and control group trabecular bones mass in spine, including BV/TV, Tb.Sp, Tb.Th and Tb.N. (DOC)

S5 Table Quantitative result of MicroCT test of BMD between diabetes group and control group trabecular bones mass in tibia, femur, mandible and spine. (DOC)
