# Supplementary material for: Different bone sites-specific response to diabetes rat models: Bone density, histology and microarchitecture
Source: PLoS One. 2018 Oct 22;13(10):e0205503. doi: 10.1371/journal.pone.0205503 (PMC6197850; doi:10.1371/journal.pone.0205503)
Supplement: S1 Table — (DOC) [file pone.0205503.s001.doc]

**Table 3:** Quantitative result of MicroCT test of diabetes group and control group trabecular bones mass in tibia, including BV/TV, Tb.Sp, Tb.Th and Tb.N.

| Tibia | | BV/TV | Tb.Sp（mm） | Tb.Th（mm） | Tb.N（1/mm） |
| --- | --- | --- | --- | --- | --- |
| 4 wks | DOP | 0.225±0.02* | 0.432±0.05** | 0.070±0.01* | 2.50±0.09** |
|  | Control | 0.309±0.02 | 0.282±0.01 | 0.103±0.01 | 3.25±0.18 |
| 8 wks | DOP | 0.162±0.02** | 0.678±0.03** | 0.059±0.01** | 2.43±0.18** |
|  | Control | 0.341±0.01 | 0.261±0.02 | 0.114±0.03 | 3.33±0.20 |
| 12 wks | DOP | 0.076±0.02** | 1.145±0.04** | 0.047±0.01** | 1.12±0.38** |
|  | Control | 0.404±0.03 | 0.247±0.25 | 0.110±0.01 | 3.69±0.40 |

Data were expressed as mean±standard deviation (SD). ^*^ p<0.05 and ^**^ p<0.01 vs. Control (ANOVA).
