# Supplementary material for: Different bone sites-specific response to diabetes rat models: Bone density, histology and microarchitecture
Source: PLoS One. 2018 Oct 22;13(10):e0205503. doi: 10.1371/journal.pone.0205503 (PMC6197850; doi:10.1371/journal.pone.0205503)
Supplement: S2 Table — (DOC) [file pone.0205503.s002.doc]

**Table 4:** Quantitative result of MicroCT test of diabetes group and control group trabecular bones mass in femur, including BV/TV, Tb.Sp, Tb.Th and Tb.N.

| Femur | | BV/TV | Tb.Sp（mm） | Tb.Th（mm） | Tb.N（1/mm） |
| --- | --- | --- | --- | --- | --- |
| 4 wks | DOP | 0.608±0.01 | 0.132±0.01 | 0.152±0.01 | 5.73±0.20 |
|  | Control | 0.614±0.05 | 0.131±0.01 | 0.159±0.02 | 5.74±0.24 |
| 8 wks | DOP | 0.606±0.03 | 0.130±0.02 | 0.16±0.01 | 5.48±0.58 |
|  | Control | 0.620±0.03 | 0.121±0.01 | 0.163±0.01 | 5.78±0.50 |
| 12 wks | DOP | 0.320±0.03** | 0.167±0.01** | 0.137±0.01** | 4.16±0.28** |
|  | Control | 0.632±0.04 | 0.128±0.01 | 0.176±0.01 | 5.75±0.41 |

Data were expressed as mean±standard deviation (SD). ^*^ p<0.05 and ^**^ p<0.01 vs. Control (ANOVA).
