# Supplementary material for: Different bone sites-specific response to diabetes rat models: Bone density, histology and microarchitecture
Source: PLoS One. 2018 Oct 22;13(10):e0205503. doi: 10.1371/journal.pone.0205503 (PMC6197850; doi:10.1371/journal.pone.0205503)
Supplement: S3 Table — (DOC) [file pone.0205503.s003.doc]

**Table 5:** Quantitative result of MicroCT test of diabetes group and control group trabecular bones mass in mandible, including BV/TV, Tb.Sp, Tb.Th and Tb.N.

| Mandible | | BV/TV | Tb.Sp（mm） | Tb.Th（mm） | Tb.N（1/mm） |
| --- | --- | --- | --- | --- | --- |
| 4 wks | DOP | 0.290±0.02* | 0.275±0.05 | 0.111±0.02 | 2.66±0.33 |
|  | Control | 0.310±0.03 | 0.226±0.02 | 0.119±0.01 | 3.53±0.27 |
| 8 wks | DOP | 0.211±0.02** | 0.352±0.03** | 0.099±0.02** | 2.39±0.18** |
|  | Control | 0.398±0.01 | 0.208±0.01 | 0.132±0.01 | 3.68±0.34 |
| 12 wks | DOP | 0.158±0.02** | 0.561±0.03** | 0.067±0.01** | 1.76±0.25** |
|  | Control | 0.494±0.02 | 0.195±0.02 | 0.170±0.02 | 3.89±0.33 |

Data were expressed as mean±standard deviation (SD). ^*^ p<0.05 and ^**^ p<0.01 vs. Control (ANOVA).
