# Supplementary material for: Different bone sites-specific response to diabetes rat models: Bone density, histology and microarchitecture
Source: PLoS One. 2018 Oct 22;13(10):e0205503. doi: 10.1371/journal.pone.0205503 (PMC6197850; doi:10.1371/journal.pone.0205503)
Supplement: S4 Table — (DOC) [file pone.0205503.s004.doc]

**Table 6:** Quantitative result of MicroCT test of diabetes group and control group trabecular bones mass in spine, including BV/TV, Tb.Sp, Tb.Th and Tb.N.

| Spine | | BV/TV | Tb.Sp（mm） | Tb.Th（mm） | Tb.N（1/mm） |
| --- | --- | --- | --- | --- | --- |
| 4 wks | DOP | 0.265±0.01 | 0.283±0.02* | 0.084±0.01 | 2.65±0.39* |
|  | Control | 0.284±0.02 | 0.230±0.03 | 0.089±0.02 | 2.71±0.33 |
| 8 wks | DOP | 0.196±0.02** | 0.387±0.03** | 0.07±0.003** | 2.22±0.37** |
|  | Control | 0.357±0.01 | 0.232±0.02 | 0.124±0.01 | 2.92±0.16 |
| 12 wks | DOP | 0.145±0.01** | 0.440±0.06** | 0.061±0.003** | 1.61±0.22** |
|  | Control | 0.404±0.02 | 0.202±0.01 | 0.142±0.02 | 3.57±0.65 |

Data were expressed as mean±standard deviation (SD). ^*^ p<0.05 and ^**^ p<0.01 vs. Control (ANOVA).
