# Supplementary material for: Different bone sites-specific response to diabetes rat models: Bone density, histology and microarchitecture
Source: PLoS One. 2018 Oct 22;13(10):e0205503. doi: 10.1371/journal.pone.0205503 (PMC6197850; doi:10.1371/journal.pone.0205503)
Supplement: S5 Table — (DOC) [file pone.0205503.s005.doc]

**Table 7：**Quantitative result of MicroCT test of diabetes group and control group trabecular bones mass in spine, including BMD.

| BMD(mg/ccm） | | Tibia | Femur | Mandible | Spine |
| --- | --- | --- | --- | --- | --- |
| 4 wks | DOP | 152±19* | 508±25 | 402±16 | 269±14 |
|  | Control | 210±20 | 520±13 | 425±20 | 287±10 |
| 8 wks | DOP | 113±8** | 500±8 | 352±14** | 172±6** |
|  | Control | 255±15 | 525±21 | 467±16 | 325±27 |
| 12 wks | DOP | 75±16** | 360±18** | 283±13** | 135±12** |
|  | Control | 308±24 | 531±18 | 485±13 | 342±20 |

Data were expressed as mean±standard deviation (SD). ^*^ p<0.05 and ^**^ p<0.01 vs. Control (ANOVA).
